# Reconstruction and *In Silico* Analysis of Metabolic Network for an Oleaginous Yeast, *Yarrowia lipolytica*

**DOI:** 10.1371/journal.pone.0051535

**Published:** 2012-12-07

**Authors:** Pengcheng Pan, Qiang Hua

**Affiliations:** State Key Laboratory of Bioreactor Engineering, East China University of Science and Technology, Shanghai, China; Virginia Commonwealth University, United States of America

## Abstract

With the emergence of energy scarcity, the use of renewable energy sources such as biodiesel is becoming increasingly necessary. Recently, many researchers have focused their minds on *Yarrowia lipolytica*, a model oleaginous yeast, which can be employed to accumulate large amounts of lipids that could be further converted to biodiesel. In order to understand the metabolic characteristics of *Y. lipolytica* at a systems level and to examine the potential for enhanced lipid production, a genome-scale compartmentalized metabolic network was reconstructed based on a combination of genome annotation and the detailed biochemical knowledge from multiple databases such as KEGG, ENZYME and BIGG. The information about protein and reaction associations of all the organisms in KEGG and Expasy-ENZYME database was arranged into an EXCEL file that can then be regarded as a new useful database to generate other reconstructions. The generated model iYL619_PCP accounts for 619 genes, 843 metabolites and 1,142 reactions including 236 transport reactions, 125 exchange reactions and 13 spontaneous reactions. The *in silico* model successfully predicted the minimal media and the growing abilities on different substrates. With flux balance analysis, single gene knockouts were also simulated to predict the essential genes and partially essential genes. In addition, flux variability analysis was applied to design new mutant strains that will redirect fluxes through the network and may enhance the production of lipid. This genome-scale metabolic model of *Y. lipolytica* can facilitate system-level metabolic analysis as well as strain development for improving the production of biodiesels and other valuable products by *Y. lipolytica* and other closely related oleaginous yeasts.

## Introduction

Nowadays with the rapidly developed world's economy, a series of problems especially energy scarcity come out and become more and more serious. As a finite and unsustainable fuel source [1], petroleum is rapidly becoming scarcer and more expensive. According to forecast, the energy demand is going to grow more than 50% by 2025 [2]. In light of these concerns, the production of renewable energy sources has become more interesting and increasingly necessary in recent years [3]. Biodiesel is one renewable energy source which gains a competitive advantage including sustainability, reduction of greenhouse gas emissions, efficiency and security of supply [4], [5]. However, the conventional production of biodiesel derived from oil crops, waste cooking oil, and animal fat usually costs too much such as land use, even worse, it has increased the cost of various food stuffs [6]. Development of novel sources of biodiesel production has therefore become more and more necessary.

Recently many researchers have been focusing their minds on oleaginous microorganisms that are able to synthesize and accumulate oil at least 25% of their dry weight and therefore act as ideal candidates for fuel production [7], [8], [9]. Among the numerous oleaginous microorganisms, *Yarrowia lipolytica* is now long used as a model organism for lipid accumulation partly because it is the only one with developed genetic tools [10].

As the only ascosporic member of the genus Yarrowia, the yeast *Y. lipolytica*, formerly known as *Candida, Endomycopsis or Saccharomycopsis lipolytica,* is often found in environments rich in hydrophobic substrates, such as alkanes, fatty acids and oils [11], [12]. Several strains of *Y. lipolytica* yeast have been isolated and can be efficiently cultivated on various substrates such as glucose (but not sucrose), alcohols and acetate. Via *de novo* synthesis pathway, this yeast is able to accumulate large amounts of lipids, in some cases to the level exceeding 50% of cell dry weight [DW] [13]. The complete genome sequence of *Y. lipolytica* was determined by B. Dujon et al. [14]. Its genome comprises more than 6000 genes in six chromosomes whose annotation was performed by the Génolevures Consortium [15]. Besides, many genetic manipulation tools on *Y. lipolytica* have been established, making rapid gene deletion and other molecular-level research possible. From the above, *Y. lipolytica* has been chosen by many researchers as the target microorganism for mechanism and regulation studies of lipid accumulation. However, even with existing tools and knowledge about *Y. lipolytica*, slow progress has been made towards understanding of lipid accumulation with classical metabolic engineering approaches.

The large quantity of information featured in public databases, like details about genomes, pathways and proteins has led to metabolic network reconstructions and *in silico* modeling of an organism's metabolic capabilities [16]. Genome-scale metabolic network (GSMN) reconstruction which represents currently available biochemical, genetic, and genomic knowledge-bases for target organisms provides a platform for omics data analysis and phenotype prediction and has become an indispensable tool for studying the systems biology of metabolism in the post-genomic era [17], [18]. Together with a variety of algorithms such as flux balance analysis (FBA) [19], [20], we can use a properly formulated genome-scale metabolic network to predict the production and optimization of an added value metabolite by target microorganism under different environmental conditions [21]. In addition, effects of environmental and genetic perturbations on the metabolic network can also be simulated [22]. Since the first genome-scale metabolic reconstruction carried on *E. coli* was released in 2000 [23], there have been up to 126 models about more than 70 organisms (http://synbio.tju.edu.cn/GSMNDB/gsmndb.htm). Though the standardized metabolic network reconstruction process has been well established [24], there are, however, only few genome-scale metabolic reconstructions about oleaginous yeasts.

In this study the metabolic network model of *Y. lipolytica* iYL619_PCP was reconstructed based on a combination of genome annotation and the more detailed biochemical knowledge. Using this metabolic reconstruction, we firstly predicted the substrates of *in silico* model and then performed gene knock-out and flux variability analysis to predict the essential genes in iYL619_PCP using glucose as the sole carbon source and help design new strains. Overall, iYL619_PCP provides a valuable tool and platform for the system-level metabolic analysis of *Y. lipolytica* and may also provide important metabolic basis and feasible engineering ways for the improvement of fatty acid synthesis in many other oleaginous yeasts.

## Materials and Methods

### Integration of information from multiple databases

Up to now, there are a large quantity of public databases that can be used to construct a genome-scale metabolic network [25]. However, information about the same organism from different databases may be varied; for example, the reaction direction may conflict. In order to construct a high-quality metabolic network for *Y. lipolytica*, information about genome, reaction and relevant protein was mined from multiple available databases, i.e. KEGG [26] and BIGG [27] (Table 1). As to the aforementioned databases, all the information about reactions and their catalyzing enzymes was downloaded from the FTP or web page of each database. Then visual BASIC for applications (VBA) was used to arrange the obtained data into EXCEL so as to be easily cited for forming genome, protein, and reaction (GPR) associations [28]. By a contrast of the genome annotation obtained from different databases, more authentic information marked with their resources (e.g., KEGG, IMG, both KEGG and IMG, etc.) was chosen and added into the draft metabolic network.

**Table 1:** Databases used in the reconstruction of metabolic network for *Y. lipolytica*.

Abbreviation	Full Name	Purpose	Link
IMG	The Integrated Microbial Genomes system	Obtain genome annotations	http://img.jgi.doe.gov/
KEGG	Kyoto Encyclopedia of Genes and Genomes	Obtain genome annotations and metabolic reactions	http://www.genome.jp/kegg/
UniProtKB	The Universal Protein resource Knowledgebase	Obtain genome annotations and metabolic reactions	http://www.uniprot.org/
ENZYME	Enzyme nomenclature database	Obtain metabolic reactions of all available enzymes	http://enzyme.expasy.org/
BIGG	Biochemical Genetic and Genomic knowledgebase	Check metabolites and reactions	http://bigg.ucsd.edu/
BRENDA		Check reactions in model	http://www.brenda-enzymes.org/

### High-quality metabolic network reconstruction of *Y. lipolytica*

**Figure 1:**
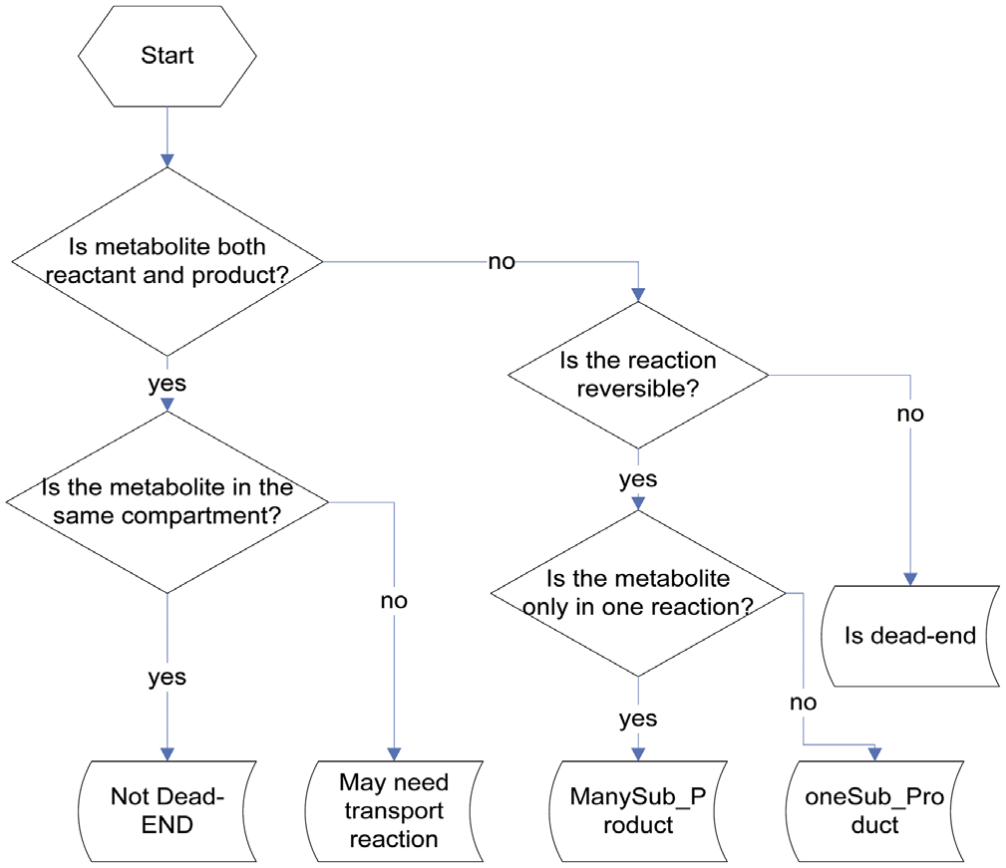
Program flow to find deadend metabolites. “oneSub_Product” represents the metabolites involved in only one reversible reaction, while “ManySub_Product” represents the metabolites participating in at least two reversible reactions. “May need transport reaction” means such metabolite can be found in multiple compartments and may need to be transported from one compartment to another.

Using the genome annotation in several public databases and information from available literature and experiment results, a genome-scale metabolic network model of *Y. lipolytica* iYL619_PCP was constructed. Because of lots of mistakes in databases such as the missing of proton in one reaction, manual curation was needed. With the help of pathway in KEGG, the draft reconstruction was refined and assembled in a pathway-by-pathway manner. In this step, we compiled programs to find the deadend metabolites (see Figure 1 for the program flow) and to validate what molecules should be added or deleted so as to balance all the reactions. With the help of available literatures and experiment results together with Brenda and ENZYME databases we also validated cofactors or direction of reactions and whether a reaction should be considered in the model system.

In our metabolic network, transport reactions for metabolites moving between compartments should be considered. However, so far transport systems of *Y. lipolytica* have not been well studied and less experimental data could be used. Here, each metabolite included in “Is deadend” category and “oneSub_Product” category (Figure 1) was studied by comparison with the transport reactions in *Saccharomyces cerevisiae* model iMM904 [29] and the flowing metabolites in our model were then determined. Besides, metabolites found to be assimilated or excreted by *Y. lipolytica* [30], [31], [32] were also included in the model by adding exchange reactions and transport reactions.

### Estimation of Biomass Composition and Maintenance Energy Requirements

An objective function, usually the formation of the biomass or the production of a target metabolite, is required to compute flux distribution in a constraint-based reconstruction [33]. The biomass compositions of *Y. lipolytica*, expressed in mmol/g DCW (dry cell weight), were calculated from various sources including published articles and available experimental data (see Additional file S6 for details). These compositions were then integrated into the genome-scale metabolic network model iYL619_PCP as a biomass synthesis reaction (also see Additional file S6) to perform flux balance analysis and flux variability analysis.

Maintenance energy accounts for the ATP requirements of various cellular processes, such as turnover of the amino acid pools and polymerization of cellular macromolecules, which can be either growth associated (GAM), i.e., related to polymerization of protein, or non-growth associated (NGAM) that is related to maintaining membrane potential. In order to estimate the maintenance requirements, experimental data obtained from continuous cell growth on glycerol was used [34]. The NGAM of *Y. lipolytica* was estimated to be 7.8625 mmol ATP/g DCW, and GAM at dilution rate of 0.1 h^−1^ was 86.7881 mmol ATP/g DCW on the assumption that a molar glycerol can be completely oxidized to generate 18.5 mol ATP.

### Flux balance analysis (FBA)

FBA [35], [36] is a constraint-based modeling approach to determine the flux distribution in genome-scale metabolic networks based on linear optimization of an objective function (typically the rate of biomass production). Accordingly, it can be used as a tool to predict the *in silico* cell growth rate or production rates of biotechnologically important metabolites such as ATP.

As mentioned above, FBA relies on the imposition of a series of constraints including equations that balance reaction inputs and outputs (Eq. 2) and inequalities that define the maximum and minimum allowable fluxes of the reactions, namely the directionality and enzymatic capacity constraints (Eq. 3). These balances and bounds imposed on model define an allowable solution space of a linear optimization (Linear Programming, LP) problem of maximize or minimize a cellular objective function such as the rate of biomass synthesis (Eq. 1) [37]. FBA corresponds to the following linear programming problem:
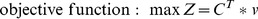
(1)

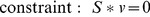
(2)

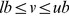
(3)where 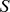 is a sparse matrix of size 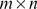, 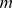 is the number of metabolites, 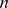 is the number of reactions, 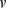 represents the flux values through all reactions, 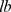 and 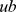 define the lowest bound and uppermost bound of each reaction respectively. In Eq. 1, 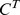 is a vector comprising the coefficient of each metabolite in the objective function.

FBA can be performed using COBRA Toolbox [38], which is a freely available Matlab toolbox. In this study, FBA was used to simulate gene deletions and analyze the assimilation of substrates and other characteristics of the model iYL619_PCP.

### Minimal media determination and substrate utilization prediction

Using FBA, we systematically predicted a minimal media composition capable of supporting growth of *Y. lipolytica*. Consulting some available articles [39], different combinations of metabolites were allowed to enter the metabolic network until the *in silico* minimal media was determined, i.e. the production of biomass is not zero. With the minimal media, each possible carbon source was allowed to flow into the *in silico* model one by one by adding exchange reactions (if there was no corresponding one) for the sake of simulating the growth of model under different environmental conditions. The results of substrate utilization prediction were compared with those obtained experimentally elsewhere [40].

### Flux variability analysis (FVA)

FVA is a frequently used computational tool to calculate the full range of numerical values for each reaction flux under a given simulation condition while maintaining some states of the network, e.g., supporting maximum biomass production rate [41]. Therefore, the maximum value 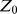 of the objective function such as biomass or ATP production was first computed with FBA described above. Then each reaction in network was set as the objective function, and the maximum and minimum flux values through each reaction were calculated with the constraints corresponding to an optimal solution 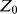 [42]. The FVA problem can be formulated as below:
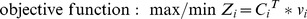
(4)

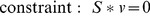
(5)

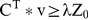
(6)

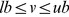
(7)where 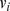 is the flux value through each reaction, both maximum and minimum flux values of a reaction were calculated to determine the full range. 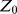 is the maximum value calculated by FBA. 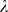, whose value is between 0 to 1, is a parameter to control whether the analysis is carried out at a suboptimal network state (when 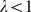) or at an optimal state (when 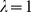).

In order to design new strains, FVA was used to measure the variation of flux of a single-gene-deleted mutant strain compared with that of the wild-type strain with the equation introduced below:
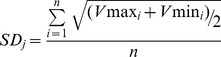
(8)

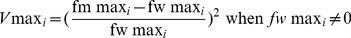
(9)

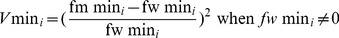
(10)where 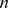 indicates the number of all reactions; 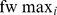 and 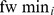 represent the maximum and minimum flux value of *i*-th reaction in the wild-type strain; 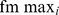 and 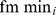 represent the maximum and minimum flux value of *i*-th reaction in the mutant strain with *j*-th gene deleted; 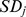 indicates the average standard deviation caused by the *j*-th gene deletion, which was used to weigh the flux variation when the *j*-th gene was deleted.

## Results

### Integration of information from multiple databases

**Figure 2:**
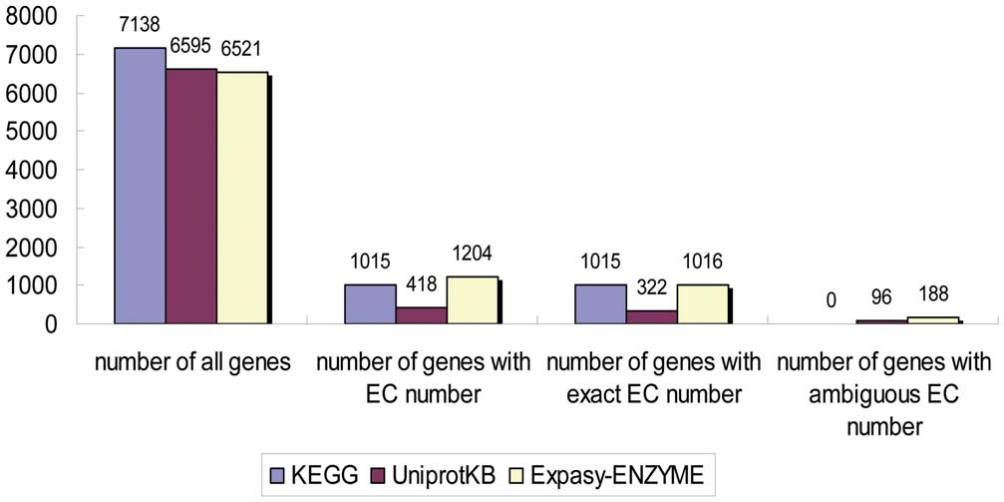
Number of genes and proteins obtained from different databases. “Genes with EC number” represents the gene that has annotation in each database, whereas “Genes with exact EC number” and “Genes with ambiguous EC number” represent the gene that has exact and ambiguous annotation in each database respectively.

Multiple databases were used to distill more accurate information about gene, protein and reaction associations of *Y. lipolytica* (Table 1). Take UniprotKB for example, the information of genes, proteins and reactions of *Y. lipolytica* was downloaded from its FTP, and the VBA codes were then compiled to store the necessary GPR associations into EXCEL (Additional file S1). The EXCEL file included information about GPR associations of *Y. lipolytica*, the genes' UniprotKB ID, the cofactor of each reaction, the pathway for each reaction and so on. All these information obtained (e.g. the most important information of GPR associations) was used to be compared with those in other databases. The number of genes and proteins obtained from each database was showed (Figure 2). As shown in this figure, there are much fewer genes with EC (Enzyme Commission) number in UniprotKB than that of any other two databases, which might be because most of the genome annotations in UniprotKB database are verified by experts. Besides, the number of genes with ambiguous EC number in KEGG is zero because the genes without exact annotations are eliminated from the FTP of KEGG.

To be worth raising, the information about protein and reaction associations of all the organisms in KEGG and Expasy-ENZYME database was collected from their FTP and stored into an EXCEL file (see Additional file S2, where some information of KEGG was updated up to Feb. 4, 2012), which would be helpful for the reconstruction of metabolic networks for other organisms.

### Genome-scale reconstruction of the metabolic network for *Y. lipolytica*

The information of different databases obtained above was compared with each other and the more correct information was chosen so as to form the draft metabolic network. With the genome annotation and other metabolic capacities in available literature and experiments, the draft metabolic network was validated and curated manually until a high-quality genome-scale metabolic network model of *Y. lipolytica* was constructed.

The resulting network, named as iYL619_PCP consists of 619 genes, 843 metabolites and 1142 reactions including 236 transport reactions and 125 exchange reactions (Table 2). Almost all the reactions except biomass reactions and exchange reactions were element-balanced and charge-balanced semi-automatically. All the elements of each metabolite in the network were arranged into EXCEL (Additional file S3) and VBA codes were compiled for the sake of automatically checking whether each reaction is balanced or not. The reversibility of each reaction was also manually validated using KEGG, Brenda database and other available articles. There are 1142 reactions in all, of which 342 reactions are non-associated with genes. Most of the reactions non-associated with genes were transport reactions (154 reactions) and exchange reactions (125 reactions). Other reactions non-associated with genes include spontaneous reactions and reactions added to fill gaps in the reconstructed network based on simulations (Additional file S3). A majority of 13 spontaneous reactions involved in iYL619_PCP are in amino acid metabolic pathway. Isolated reactions and repeated reactions were eliminated from the metabolic network (Additional file S3). 551 out of 843 metabolites participate in 761 cytoplasmic reactions.

**Table 2:** Basic properties of metabolic network iYL619_PCP.

Features	number
Compartment	3
genes	619
reaction	1142
Associated with genes	800
Not associated with genes	342
Transport reactions	236
Exchange reactions	125
Extracellular reactions	239
Cytoplasmic reactions	761
Mitochondrial reactions	142
Metabolic reactions	781
Reversible reactions	563
Irreversible reactions	218
Spontaneous reactions	13
Metabolites	843
Extracellular metabolites	128
Cytoplasmic metabolites	551
Mitochondrial metabolites	164

**Figure 3:**
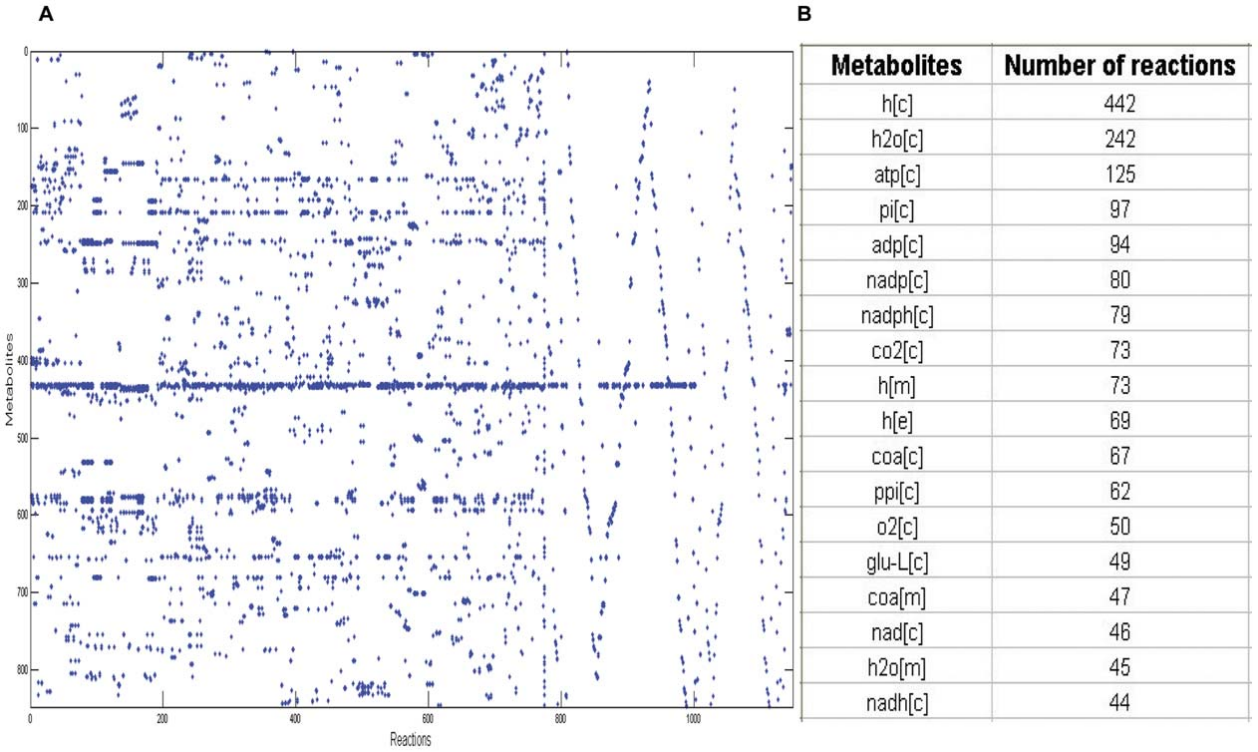
Analysis of the S matrix of the model iYL619_PCP. (A) The visualization of the (843*1142) S matrix of the model iYL619_PCP, (B) Most connected metabolites in the iYL619_PCP metabolic network.

More information of the model iYL619_PCP can be found in Additional file S3. Due to the limited knowledge about *Y. lipolytica*, there were still 60 dead-ends included in 59 reactions in the model iYL619_PCP (Additional file S3). As described above, 843 metabolites within 1142 reactions in the model can be shown in the form of S matrix (843*1142). The *Y. lipolytica* model S matrix was visualized using Matlab, where all non-zero entries in S were represented with a dot, as shown in Figure 3 (A). Many metabolites were shown with a large number of dots, indicating their participation in many metabolic reactions. Figure 3 (B) listed several metabolites and numbers of participating reactions, where the metabolite proton is related to 442 cytoplasmic reactions in the model iYL619_PCP, suggesting 442 dots in the row of proton in Figure 3 (A). From the network topology perspective, all the metabolites in iYL619_PCP displayed the connectivity distribution patterns similar to that of the other microbial genome-scale networks, such as yeast [43]. A small quantity of metabolites participate in very many reactions, whereas most of the metabolites have few connections. Most connected metabolites include the current metabolites (e.g. ATP, NADPH, NADH), key metabolites in the central metabolism (e.g. pyruvate, succinate, fructose-6-phosphate), a couple of amino acids and its precursors such as L-glutamate, and key metabolites and precursors in the lipid biosynthesis pathway (e.g. malonyl-ACP, ACP, acetyl-CoA). These most connected metabolites play important roles in the metabolic network, for example, the stability in transporting such highly connected metabolites into or out of the network will affect globally the *in silico* metabolic phenotype. In addition, the organization of regulatory mechanisms may be significantly affected by such highly connected metabolites. In order to discover the corresponding regulatory mechanisms, more and more researchers concentrate their attentions on these highly connected metabolites. Furthermore, the connection of metabolites can be found in Additional file S3.

### Minimal media determination

Using the method mentioned above an *in silico* minimal media composition capable of supporting growth of *Y. lipolytica* was systematically predicted with the help of available literatures that revealed experimental growth requirements (see Additional file S4). The glucose minimal media composition is shown in Table 3. By using the FBA method and this minimal media, a maximum flux of 0.0439 was predicted for cells growing in glucose minimal media where glucose uptake rate and ammonium consumption rate were assumed to be 20mmol/(g DCW·h) and 3 mmol/(g DCW·h) respectively.

**Table 3:** The *in silico* glucose minimal media composition of the model iYL619_PCP.

Reaction name	Reaction description	Equation	LB* ^1^	UB* ^2^
R1294	D-glucose exchange	[e]: D-glucose < = >	−20	1000
R1196	Sulfate exchange	[e]: so_4_ < = >	−1000	1000
R1204	O_2_ exchange	[e]: o_2_ < = >	−1000	1000
R1211	Phosphate exchange	[e]: pi < = >	−1000	1000
R1218	Ammonia exchange	[e]: nh_4_ < = >	−1000	1000
R1221	H_2_O exchange	[e]: h_2_o < = >	−1000	1000
R1228	CO_2_ exchange	[e]: co_2_ < = >	−1000	1000
R1305	Proton exchange	[e]: h < = >	−1000	1000

* 1, LB, lower bound, whose unit is mmol/(gDW*h^−1^).

* 2, UB, upper bound, whose unit is also mmol/(gDW*h^−1^).

**Figure 4:**
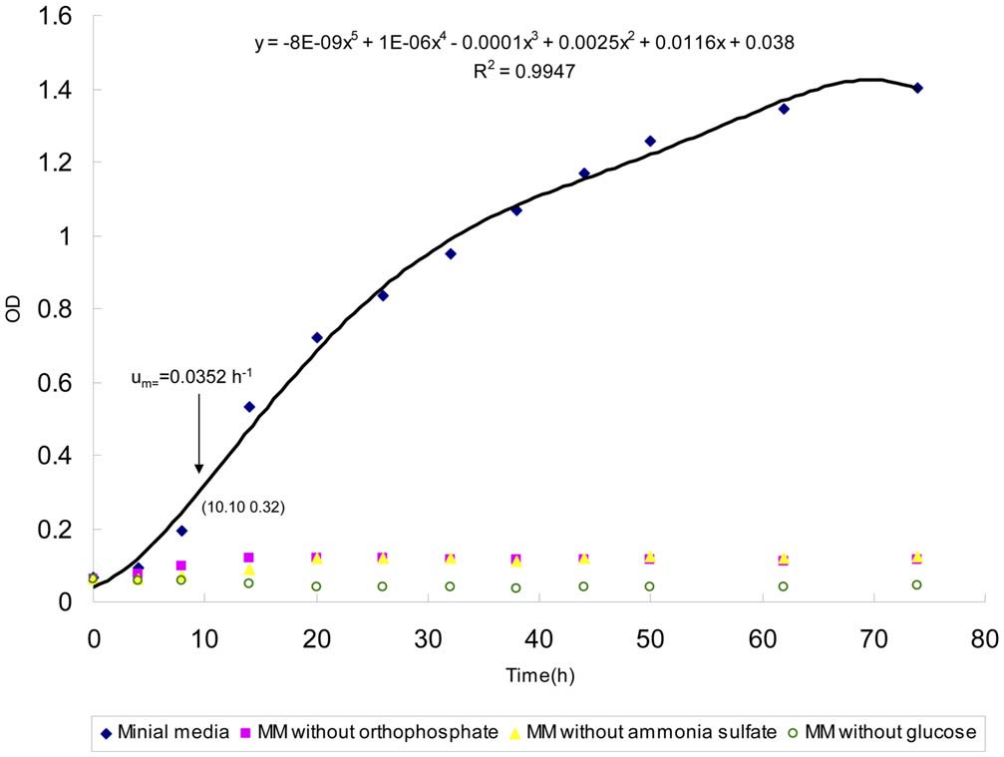
Experimental validation of the minimal media predicted in silico with the model iYL619_PCP. The composition of minimal media in experiment is as follows: glucose (20g/L), NH_4_SO_4_ (3g/L), KH_2_PO_4_(2g/L). The OD of culture media after inoculation was about 0.06.

In addition, the flask growth of the wild-type strain of *Y. lipolytica* in the minimal media (Table 3) was experimentally tested, during which optical density (OD) was detected with spectrophotometer. The results shown in Figure 4 indicated that *Y. lipolytica* could grow on this minimal media with a maximum specific growth rate of approximately 0.0352 h^−1^, which is consistent with the predicted value. In addition, no growth of *Y. lipolytica* was observed with the absence of ammonia sulfate, glucose or orthophosphate in the media (see Figure 4).

### Substrate utilization validation of *in silico* model iYL619_PCP

Utilization of various substrates by *Y. lipolytica* has been reported previously by Kurtzman [40]. We compared the *in silico* prediction on the use of substrates with available experimental data (Table 4). During this process, in which FBA was used, twenty-nine substrates were chosen to predict whether *Y. lipolytica* can grow on each substrate with the minimal media defined above. As shown in Table 4, the growth on twenty-four out of twenty-nine substrates were correctly predicted, resulting in a predictive accuracy of about 83%. The wrongly predicted instances included GN (substrate that can be assimilated in experiment but can't *in silico*) and NG (substrate that can't be assimilated in experiment but can *in silico*). There are four GN-substrates including D-mannitol, D-glucitol, succinate and hexadecane and only one NG-substrate of trehalose. These instances of GN indicate that the model iYL619_PCP has a weaker ability than that it should have, whereas the instance of NG just in opposite, indicate the model has a stronger ability. There might be at least two reasons for four GN-substrates in the model iYL619_PCP. On one hand, some reactions associated with the assimilation of these four substrates were overlooked, resulting in gaps in biosynthetic pathways. On the other hand, the equation of biomass reaction might be responsible for four wrong predictions. For example, if a component was not a part of biomass but was wrongly included into the biomass equation, the predicted biomass synthesis flux will be zero in case that this component can not be synthesized from a certain carbon source. This implied that further validation and improvement of biomass compositions are required for the constructed model system. Apropos of trehalose, the only NG-substrate, a reaction catalyzed by the product of gene YALI0D15598g was found responsible for the synthesis of glucose from trehalose. Meanwhile, by referring to some articles [44], some regulation mechanisms of trehalose metabolism were found, whereas there were no mechanisms included in the model iYL619_PCP. So the possible reason for the discrepancy between experimental and simulated growth on trehalose might be some wrongly added reactions or missing of regulation network in the model iYL619_PCP. The current model iYL619_PCP therefore needs to be improved with possible regulatory mechanisms and further curated.

**Table 4:** Comparison of substrate utilization between experimental and *in silico* data.

Substrate	Experiment	Prediction	Substrate	Experiment	Prediction
Glucose	+	+	Hexadecane	+	–
N-Acetyl-D-glucosamine	+	+	Sucrose	–	–
Methanol	–	–	Maltose	–	–
Ethanol	+	+	Cellobiose	–	–
Glycerol	+	+	Trehalose	–	+
Galactitol	–	–	Lactose	–	–
D-Mannitol	+	–	Melibiose	–	–
D-Glucitol	+	–	Raffinose	–	–
Mehyl-D-glucoside	–	–	Inulin	–	–
Salicin	–	–	D-xylose	–	–
D-Lactate	+	+	L-Arabinose	–	–
L-Lactate	+	+	D-Arabinose	–	–
Succinate	+	–	L-Rhamnose	–	–
Citrate	+	+	D-Glucosamine	–	–
Inositol	–	–			

“+” means the substrate can be assimilated.

“–“ means the substrate can not be assimilated.

### Gene deletion analysis

**Figure 5:**
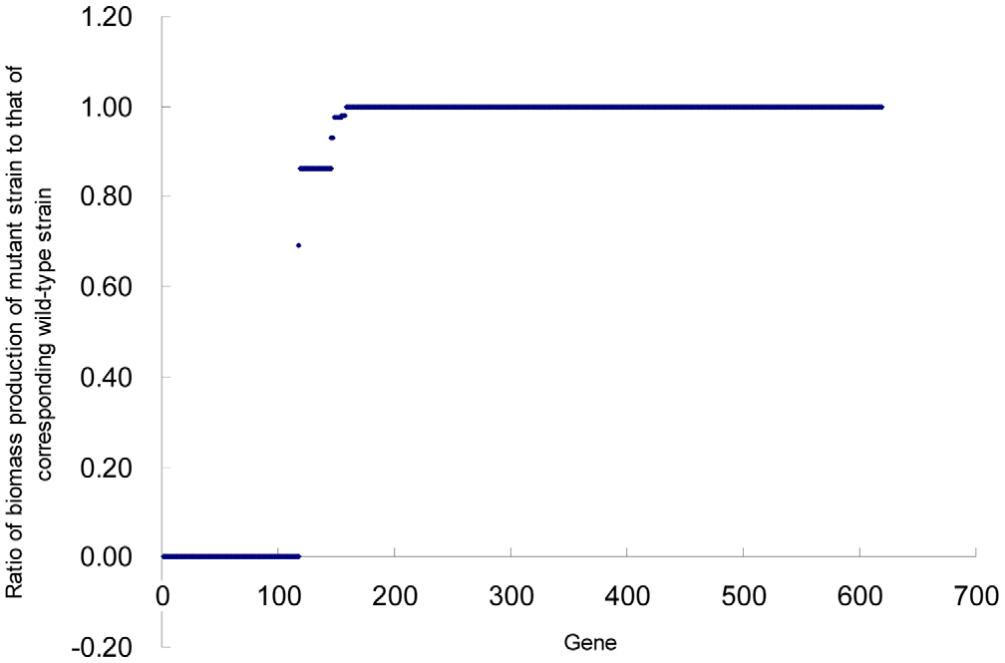
Results of single gene deletion study. The horizontal axis represents every gene in the metabolic model; the vertical axis represents the ratio of biomass production of a single gene-deletion strain to that of the wild-type strain.

In practical experiments, genetic manipulation has become an indispensable tool to design mutant strains and validate phenotypic outcomes. However, the process of gene deletion is very time-consuming. In order to reduce the scope of experiments and quickly investigate essentiality of each gene in *Y. lipolytica*, a single gene deletion study with FBA was conducted with model iYL619_PCP in the glucose-minimal media described above. As shown in Figure 5, the results could be divided into three categories: i) 117 essential genes [45], deletion of which completely inhibits biomass growth, ii) 51 partially essential genes, deletion of which affects but does not completely inhibit biomass growth, iii) 451 non-essential genes, deletion of which exhibits no effect on biomass growth. More information of the prediction can be obtained from Additional file S4. Though gene essentiality is related to environmental condition and biomass reaction, the results obtained in this study might provide significant potential use and guidance for the metabolic engineering of *Y. lipolytica*.

### Flux variability analysis for strain design

**Figure 6:**
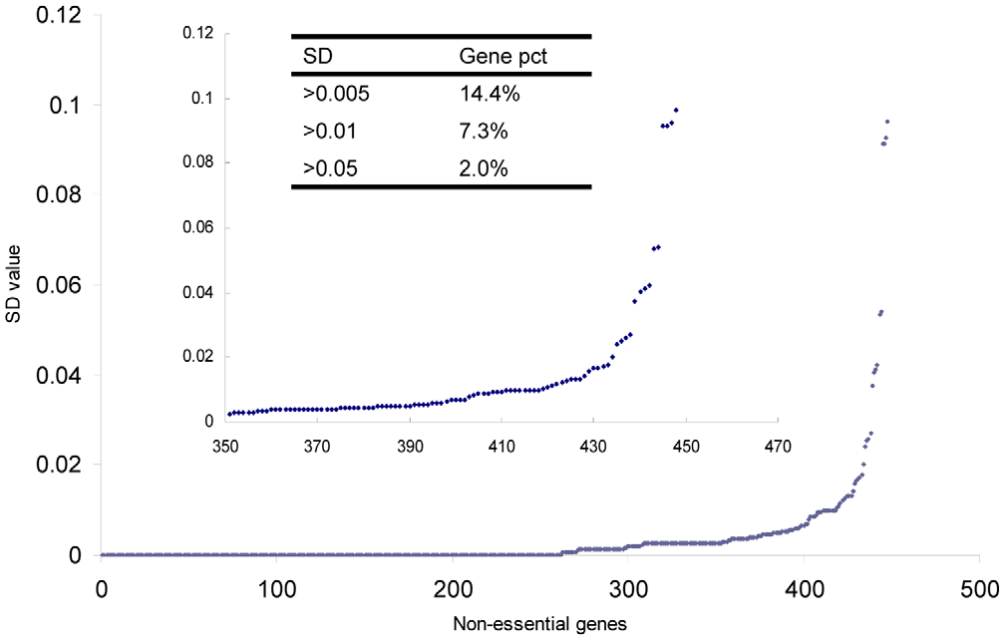
The SD values calculated of all non-essential genes. “SD” represents the average standard deviation calculated using Eq. 8, “Gene pct” represents the percent of SD values in each range, because SD values of some non-essential genes can’t be calculated, SD values of such type are set as zero and showed here.

Flux variability analysis was used to help design strains that can produce more lipids. Because lipids such as triglyceride are intracellular products, the production of lipids is in great relation to biomass production. Here in order to help design mutant strains that produce a large mount of lipids, the biomass production was set at the optimal value 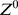 (described above) until the influencing factor of lipid production was experimentally studied clearly. FVA was used to evaluate the effects of individual non-essential genes on flux distribution (see Additional file S4 for the flux distribution of each reaction), and the SD value of each non-essential gene was calculated and shown in Figure 6 (the SD values of three genes were too high and not shown here, see Additional file S4 for more details). Only a few non-essential genes exhibited large SD values, indicating great effects of these genes on overall flux distribution. To perform gene manipulation and strain engineering more efficiently, the scope of genes could be restricted to these genes with larger SD values. For example, with the above definition of SD value, the deletion of gene “YALI0C11407g” resulted in the largest SD value (Additional file 4), indicating that this gene might have the greatest effects on overall flux distribution and could be regarded as a candidate for engineering. In the model, the “YALI0C11407g” gene encodes acetyl-CoA carboxylase that catalyzes the conversion of acetyl-CoA to mal-CoA (reaction “R0162”). Interestingly, literature information [46], [47] shows that acetyl-CoA carboxylase is responsible for the control of rate-limiting step of de novo fatty acids synthesis. It therefore provides evidence that the FVA-based analysis is informative and the gene deletions with large SD values probably have large impacts on the decision of an engineering strategy. In case that the maximum or minimum flux value of the *i*-th reaction in the wild-type strain was zero, 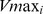 and 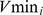 could not be calculated by the equations above (Eq. 9 and Eq. 10). However, if the i-th reaction is closely related to a certain target product and the deletion of the *j*-th gene resulted in a non-zero flux of the *i*-th reaction in the mutant strain, the *j*-th gene might be a good candidate for strain engineering to achieve improved yield of the target product. For example, reaction “R0272” shows that serine is synthesized from 3-Phosphoglycerate. FVA results indicated that the minimum flux value of this reaction was zero in the wild-type strain, whereas the knockout of either gene “YALI0F16819g” or gene “YALI0F09185g” resulted in a minimum flux of 19.7162 mmol/g DCW/h and 19.6913 mmol/g DCW/h respectively. Although in this example 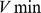 could not be calculated with Eq. 9, both genes could be considered as good engineering targets for enhanced serine synthesis. In fact, the “YALI0F16819g” gene encodes the enzyme converting 2-phospho-D-glycerate to phosphoenolpyruvate and the product of “YALI0F09185g” catalyzes the conversion of phosphoenolpyruvate to pyruvate. A deficiency of either enzyme might result in the accumulation of 3-Phosphoglycerate and thus enhance the flux of serine synthesis. Table 5 shows parts of the above reaction-gene pairs obtained from FVA (see Additional file S4 for more details), from which specific genes are suggested to be engineered to obtain certain enhanced metabolic functions.

**Table 5:** Reactions with zero flux in the wild-type strain but non-zero flux when the corresponding gene was deleted.

Group I *		Group II **	
Reaction	Gene	Reaction	Gene
R0133	YALI0D17864g	R0635	YALI0C23210g
R0135	YALI0D17864g	R0287	YALI0F09185g or YALI0F16819g
R0146	YALI0C05951g	R0512	YALI0F30129g
R0148	YALI0C05951g		
R0154	YALI0D17864g		
R0155	YALI0D17864g		
R0272	YALI0F09185g or YALI0F16819g		
R0275	YALI0F05874g		
R0309	YALI0D10813g		
R0310	YALI0E16797g or YALI0F19514g		
R0311	YALI0D07986g		

* Group I: reaction with zero minimum flux in the wild-type strain, but non-zero minimum flux when the corresponding Group I gene was deleted.

** Group II: reaction with zero maximum flux in the wild-type strain, but non-zero maximum flux when the corresponding Group I gene was deleted.

## Discussion

We have successfully reconstructed a genome-scale metabolic network model iYL619_PCP for *Y. lipolytica* with the help of available knowledge in public databases and scientific publications. The model iYL619_PCP is the first genome-scale metabolic reconstruction for oleaginous yeasts with information directly derived from multiple databases. Both the SBML representation (see Additional file S5) and the EXCEL representation of the model are freely available so that it provides a basis for the improvement of reconstructions for oleaginous yeasts. During the reconstruction of iYL619_PCP, the genome annotations in KEGG, IMG, UniprotKB were picked up and compared automatically to decide which annotation should be added into the metabolic network. Besides, the information about protein and reaction associations of all the organisms in KEGG and Expasy-ENZYME databases was obtained and stored into an EXCEL file, which therefore provides a platform to generate other reconstructions.

The model iYL619_PCP as a chemically and genetically structured database comprises 619 genes, 843 metabolites and 1142 reactions including 236 transport reactions, 125 exchange reactions and 13 spontaneous reactions. Because of the lack of knowledge of gene annotations in several public databases, 78 reactions without gene associations were added into the model to satisfy the production of biomass and other characters of *Y. lipolytica*. The deadends were defined and 60 deadends in 59 reactions were found using a VBA program. In iYL619_PCP almost all the reactions were element-balanced and charge-balanced, and the names of metabolites in model followed the conventional naming rule [48] and their molecular formation and charge were all ensured one by one, which will facilitate balancing the reactions.

Before we presented our metabolic model iYL619_PCP, another model iNL895 for *Y. lipolytica* [49] was on-line published in the journal BMC system biology. The reconstruction of iNL895 was derived from the models of a phylogenetically distant yeast *S. cerevisiae*, whereas iYL619_PCP was derived directly from the well-known databases with more credible information of *Y. lipolytica*. There are some differences in the information included in iYL619_PCP and iNL895, so the two models can complement each other in the future metabolic study and engineering of this or other related oleaginous yeasts.

The *in silico* minimal media of the model was ensured and experimentally tested, with which the utilization of 29 substrates was predicted and compared with the results reported elsewhere. The growths of *Y. lipolytica* on 24 substrates were correctly predicted, indicating the model iYL619_PCP could be used to qualitatively predict the production of biomass under various substrate conditions. Besides, four GN-substrates and one NG-substrate were identified, which could be used to direct the improvement of the model system. A single gene deletion study of the model iYL619_PCP was performed in the glucose-minimal media, and 117 essential genes, 51 partially essential genes and 451 non-essential genes were identified, which will be partially verified experimentally in our future study and now provides a guidance for genetic manipulations of *Y. lipolytica*. Using FVA, the model iYL619_PCP was used to design mutant strain. The effects of individual non-essential genes on overall flux distribution were evaluated with the introduction of novel average standard deviation SD, which can therefore be used to reduce the scope of genetic manipulation for the design novel mutant strains for enhanced production of lipids and some useful chemicals.

The genome-scale metabolic model iYL619_PCP could be employed for the prediction of certain physiological and metabolic functions. However, due to the lack of sufficient knowledge of *Y. lipolytica*, this model still needs iterative curation, for example, the prediction of essential genes may be not accurate enough, and as a result of the lack of regulatory mechanisms, some predictions such as the assimilation of trehalose might disagree with the experimental results. We expect in future the current model of iYL619_PCP could largely help researchers to improve the strain and experimental designs and in turn, the growth experimental evidence will significantly benefit the improvement of GPR associations and prediction accuracy of the genome-scale model iYL619_PCP.

## Supporting Information

Additional file S1 Genes and genome annotations of *Yarrowia lipolytica* downloaded from KEGG, UniprotKB and IMG databases.
(XLSX)

Additional file S2 A database of all protein and reaction associations sorted from KEGG and Expansy-Enzyme.
(XLSX)

Additional file S3 The reconstructed genome-scale metabolic model iYL619_PCP (XLS), including metabolite list, genome annotation, detail of reactions and metabolites, eliminated reactions and added reactions during the curation and some characters of the model.
(XLSX)

Additional file S4 Results of flux balance analysis and flux variability analysis and information used to help determine the *in silico* minimal media.
(XLSX)

Additional file S5 The model iYL619_PCP in SBML format
(XML)

Additional file S6 Determination of biomass composition and estimation of energy requirements of *Yarrowia lipolytica.*
(DOC)
