# Supplementary material for: Reconstruction and In Silico Analysis of Metabolic Network for an Oleaginous Yeast, Yarrowia lipolytica
Source: PLoS One. 2012 Dec 7;7(12):e51535. doi: 10.1371/journal.pone.0051535 (PMC3518092; doi:10.1371/journal.pone.0051535)
Supplement: Additional file S6 — Determination of biomass composition and estimation of energy requirements of Yarrowia lipolytica. (DOC) [file pone.0051535.s006.doc]

**Biomass composition and energy requirements of *Yarrowia lipolytica***

This file contain the the tables of detailed macromolecular composition of a gram of *Yarrowia lipolytica.* Besid*e*s, the energy requirements was compulated according the obtained information.

Table of Contents

[Table 1. Overall Macromolecular composition of *Y. lipolytica.* 2](#__RefHeading___Toc334720793)

[Table 2. Amino acid composition of *Y. lipolytica*. 2](#__RefHeading___Toc334720794)

[Table 3. RNA Composition of 1 Gram of *Y. lipolytica* Cell 3](#__RefHeading___Toc334720795)

[Table 4. DNA Composition of 1 Gram of *Y. lipolytica* Cell. 4](#__RefHeading___Toc334720796)

[Table 5. Carbohydrate composition of *Y. lipolytica* 4](#__RefHeading___Toc334720797)

[Table 6. Fat acid composition of *Y. lipolytica*. 4](#__RefHeading___Toc334720798)

[Table 7. Lipid composition of *Y. lipolytica*. 4](#__RefHeading___Toc334720799)

[Table 8. Neutral lipids composition of *Y. lipolytica* 5](#__RefHeading___Toc334720800)

[Table 9. Detailed lipid composition of *Y. lipolytica*. 5](#__RefHeading___Toc334720801)

[Table 10. Phospholipid content of *Y. lipolytica.* 6](#__RefHeading___Toc334720802)

[Table 11. Cellular components of *Y. lipolytica.* 6](#__RefHeading___Toc334720803)

[Table 12. ATP requirements for biosynthesis of cell polymers during growth on glucose. 7](#__RefHeading___Toc334720804)

To determine a detailed list of the cellular components of *Y. lipolytica*, many articles and related reports were checked to find useful information of the composition of biomass.

Table 1. Overall Macromolecular composition of *Y. lipolytica.*

| Component | Cellular content% (w/w) | References and comments |
| --- | --- | --- |
| Protein | 35.00 | [1] |
| Carbohydrates | 11.03 | [1] |
| RNA | 6.30 | [2] |
| DNA | 3.16 | [3,4] |
| Lipid | 40.00 | [1] |
| Ash | 5.00 | [1] |
| Sum | 100.49 |  |

In order to compute the fraction of DNA in dry cell, the information of the mass and volume of a cell in the articles[3,4] were used. The computing process is as follows:

$\text{The mass of DNA in a cell }=\text{ 36.8 fg}$[4]

$\text{The volume of a cell }=\frac{186.0448}{48}\mu m^{3}=3.8759\mu m^{3}$[3]

So if we assume cell density is equal to the density of water which is 1 g/ml and a cell has 70% water[5], the fraction of DNA in a dry cell $=\frac{36.8\times10^{-15}g}{1\times3.8759\times10^{-12}g\times(1-0.7)}=3.1648\%$

Table 2. Amino acid composition of *Y. lipolytica* (The first two columns were adopted from [6]).

| Amino acid | composition %(molar fraction) | MW (g/mol)^*^ | Correct MW (g/mol)^**^ | Weight g (if total 1 mol amino acid) | Content  mmol/g DCW |
| --- | --- | --- | --- | --- | --- |
| ala-L | 8.1 | 89 | 71 | 5.751 | 0.247 |
| arg-L | 4.8 | 175 | 157 | 7.536 | 0.146 |
| asn-L | 5.4 | 132 | 114 | 6.156 | 0.165 |
| asp-L | 5.4 | 132 | 114 | 6.156 | 0.165 |
| cys-L | 1 | 121 | 103 | 1.03 | 0.031 |
| gln-L | 6.6 | 146 | 128 | 8.448 | 0.201 |
| glu-L | 6.6 | 146 | 128 | 8.448 | 0.201 |
| gly | 5.9 | 75 | 57 | 3.363 | 0.180 |
| his-L | 3.1 | 155 | 137 | 4.247 | 0.095 |
| ile-L | 4.4 | 131 | 113 | 4.972 | 0.134 |
| leu-L | 8.8 | 131 | 113 | 9.944 | 0.268 |
| lys-L | 7 | 147 | 129 | 9.03 | 0.214 |
| met-L | 3.1 | 149 | 131 | 4.061 | 0.095 |
| phe-L | 4.8 | 165 | 147 | 7.056 | 0.146 |
| pro-L | 4.7 | 115 | 97 | 4.559 | 0.143 |
| ser-L | 5.6 | 105 | 87 | 4.872 | 0.171 |
| thr-L | 6.1 | 119 | 101 | 6.161 | 0.186 |
| trp-L | 1 | 204 | 186 | 1.86 | 0.031 |
| tyr-L | 3.1 | 181 | 163 | 5.053 | 0.095 |
| val-L | 6.1 | 117 | 99 | 6.039 | 0.186 |
| sum | 101.6 |  |  | 114.742 | 3.099 |

^*^ Molecular weight was calculated from the elementary composition, data of about cystine and tryptophan was not included in article [6], assume their molecular fraction in the protein are both 1%.

^**^ During protein synthesis, every amino acid will take a water molecule off, so the correct molecular weight can be obtained by Molecular weight minus the molecular weight of water which is 18g/mol.

The content(mmol/g DCW) of each amino acid can be computed, take Alanine as an example,

$$\text{composition (weight fraction)}=\frac{5.751}{114.742}(g/g\Pr otein)=5.012\%$$

$$\text{content(mmol/gDCM)}=\frac{\text{5.012\%}\times0.35}{71}\times1000g/gDM=0.247mmol/gDW$$

Table 3. RNA Composition of 1 Gram of *Y. lipolytica* Cell (The first two columns were adopted from [7]).

| metabolite | composition (molar fraction) | MW (g/mol) ^*^ | Weight g (if total 1 mol RNA) | Content  mmol/g DCW^**^ |
| --- | --- | --- | --- | --- |
| cmp | 0.20000 | 323 | 64.6 | 0.0368 |
| gmp | 0.32222 | 363 | 116.9667 | 0.0593 |
| ump | 0.21587 | 324 | 69.94286 | 0.0397 |
| amp | 0.26190 | 347 | 90.88095 | 0.0482 |
| Sum | 100 |  | 342.3905 |  |

^*^ Molecular weight was calculated from the elementary composition.

^**^ The method of computing the composition (molar fraction) of each part of RNA is the same as that of amino acid.

Table 4. DNA Composition of 1 Gram of *Y. lipolytica* Cell (GC content of *Y. lipolytica* is 49.6%[8]).

| metabolite | composition (molar fraction) | MW (g/mol) ^*^ | Weight g (if total 1 mol RNA) | Content  mmol/g DCW^**^ |
| --- | --- | --- | --- | --- |
| damp | 0.252 | 331 | 83.412 | 0.0244 |
| dcmp | 0.248 | 307 | 76.136 | 0.0240 |
| dgmp | 0.248 | 347 | 86.056 | 0.0240 |
| dtmp | 0.252 | 322 | 81.144 | 0.0244 |
| Sum | 1 |  | 326.748 |  |

^*^ Molecular weight was calculated from the elementary composition.

^**^ The method of computing the composition (molar fraction) of each part of DNA is the same as that of amino acid.

Table 5. Carbohydrate composition of *Y. lipolytica* (The first two columns were adopted from [1]).

| Carbohydrate | content in dry cell(mg/g) | MW (g/mol) ^*^ | mmol/gDCW |
| --- | --- | --- | --- |
| chitin(monomer) | 52.6 | 203 | 0.259113 |
| Trehalose | 0.7 | 342 | 0.002047 |
| Mannan | 11.4 | 162 | 0.07037 |
| glucan | 45.6 | 162 | 0.281481 |

^*^ Molecular weight was calculated from the elementary composition.

Table 6. Fat acid composition of *Y. lipolytica* (The first two columns were adopted from [9]).

| Fatty Acid | Content %(w/w) | MW (g/mol) ^*^ | Content (mol/g fat acid) | Content (mol/mol fat acid) | Average MW (g/mol fat acid) |
| --- | --- | --- | --- | --- | --- |
| C12:0 | 0.3 | 199 | 0.001507538 | 0.004352773 | 0.866202 |
| C14:0 | 0.2 | 227 | 0.000881057 | 0.002543912 | 0.577468 |
| C16:0 | 19 | 255 | 0.074509804 | 0.215135114 | 54.85945 |
| C18:0 | 4.2 | 283 | 0.014840989 | 0.042850978 | 12.12683 |
| C18:1 | 51.5 | 281 | 0.183274021 | 0.529174356 | 148.698 |
| C18:2 | 19.9 | 279 | 0.071326165 | 0.205942867 | 57.45806 |
| Sum | 95.1 |  | 0.346339575 | Average | 274.586 |

^*^ Molecular weight was calculated from the elementary composition.

Table 7. Lipid composition of *Y. lipolytica* (The first two columns were adopted from [10]).

| Lipid | Composition %(w/w in dry cell) |
| --- | --- |
| N(Neutral lipids) | 90 |
| G+S(glycolipids plus sphingolipids) ^*^ | 2.2 |
| P(phospholipids) | 7.8 |
| sum | 100 |

^*^ glycolipids plus sphingolipids were not included in the model.

Table 8. Neutral lipids composition of *Y. lipolytica* (adopted from[11]).

| Neutral lipid | Composition %(w/w in Neutral lipids) |
| --- | --- |
| TAG(Triacylglycerol) | 84.5 |
| E(ergosterol) | 0.6 |
| EE（ergosteryl ester）^*^ | 5.1 |
| SE(steryl esters:zymosterol) | 2.7 |
| P(phospholipids) | 2 |
| Totle | 94.9 |

^*^ ergosteryl ester was not included in the model.

According to Table 7 and Table 8, the lipid composition of *Y. lipolytica* can be obtained as Table 9.

Table 9. Detailed lipid composition of *Y. lipolytica*.

| Lipid | Content %(w/w Lipid) | MW (g/mol) | Content (mmol/gDCW) |
| --- | --- | --- | --- |
| TAG(Triacylglycerol) | 76.05 | 912.7580 | 0.333275628 |
| E(ergosterol) | 0.54 | 396.6484 | 0.005445629 |
| SE(steryl esters : zymosterol) | 2.43 | 384.6377 | 0.025270534 |
| P(phospholipids) | 9.6 |  |  |

As to the molecular weight of triacylglycerol, the core structure has a molecular weight of 89 g/mol, besides, it also has three fatty acid chains attach to the core structure, so average molecular weight of triacylglycerol is 912.758 g/mol (i.e. 89+3*274.586). The content of phospholipids was (7.8+90*0.02)%, which is 9.6%.

For instance for triacylglycerol,

$$\text{Content (mmol/gDW)}=\frac{\text{76.05\%}\times\text{40\%}}{912.7580}\times1000mmol/gDW=\text{0.333275628mmol/gDW}$$

For phospholipids, because there is no corresponding information published, so the information of the other yeast Saccharomyces cerevisiae was used(the first two columns) [12].

Table 10. Phospholipid content of *Y. lipolytica.*

| Phospholipids | phospholipids (%) | Avg MW (g/mol) | Content (mmol/gDCW) |
| --- | --- | --- | --- |
| PA (Phosphatidate) | 2.5 | 719.172 | 0.001334868 |
| PINS (phosphatidyl-1D-myo-inositol) | 28.7 | 881.172 | 0.012506979 |
| PS | 8 | 806.172 | 0.003810601 |
| PE | 20.5 | 762.172 | 0.010328377 |
| PC | 29.2 | 794.172 | 0.014118856 |

The average molecular weight of fat acids was also used to determine the average molecular weight of phospholipids.

For instance for phosphatidate,

$$\text{Content (mmol/gDW)}=\frac{\text{2.5\%}\times\text{9.6\%}\times\text{40\%}}{719.172}\times1000mmol/gDCW=\text{0.001334868mmol/gDCW}$$

The overall cell composition is therefore as follows:

Table 11. Cellular components of *Y. lipolytica.*

| Metabolite | Content (mmol/gDCW) | Metabolite | Content (mmol/gDCW) |
| --- | --- | --- | --- |
| 13BDglcn | 0.2815 | ile-L | 0.1342 |
| ala-L | 0.2471 | leu-L | 0.2684 |
| amp | 0.0482 | lys-L | 0.2135 |
| arg-L | 0.1464 | mannan | 0.0704 |
| asn-L | 0.1647 | met-L | 0.0946 |
| asp-L | 0.1647 | pa_YL | 0.0013 |
| cmp | 0.0368 | pc_YL | 0.0141 |
| cys-L | 0.0305 | pe_YL | 0.0103 |
| damp | 0.0244 | phe-L | 0.1464 |
| dcmp | 0.0240 | pro-L | 0.1434 |
| dgmp | 0.0240 | ps_YL | 0.0038 |
| dtmp | 0.0244 | ptd1ino_YL | 0.0125 |
| ergst | 0.0054 | ser-L | 0.1708 |
| gln-L | 0.2013 | so_4_ | 0.020 |
| glu-L | 0.2013 | thr-L | 0.1861 |
| gly | 0.180 | tre | 0.002 |
| Chitin | 0.2591 | triglyc_YL | 0.3333 |
| gmp | 0.0593 | trp-L | 0.0305 |
| zymst | 0.0253 | tyr-L | 0.0946 |
| his-L | 0.0946 | ump | 0.0397 |
| val-L | 0.061 |  |  |

Table 12. ATP requirements for biosynthesis of cell polymers during growth on glucose.

|  | Cellular content %(w/w) | Per Molecule (mmol ATP/g polymer) | Total(mmol ATP/ g cell) |
| --- | --- | --- | --- |
| Protein | 35 | 41.5 | 14.525 |
| Carbohydrates | 11.03 | 12.8 | 1.28 |
| RNA | 6.3 | 26 | 1.638 |
| DNA | 3.16483 | 26 | 0.8228558 |
| Lipid | 40 | 25.6 | 10.24 |
| Sum |  |  | 28.6376958 |

The estimate of ATP requirements for biosynthesis of cell polymers during growth on glucose is low than the true value[13]. The GAM and NGAM of *Y. lipolytica* growing on glycerol in a continuous culture[14] were computed and added into the model.

Assume a molar glycerol can be completely oxidized and 1 molar glycerol can generate 18.5 molar ATP. According to the article[14], the representation of specific rates of glycerol consumption(q_Glol_ g/(g•h)) versus the dilution rate (D h^-1^) in a single-stage continuous fermentation by *Y. lipolytica* is as follows:

$$q_{Glol}=0.034+3.753D$$

So $NGAM=\frac{0.034}{80}\times18.5\times1000mmol/(gDCW\bullet h)=7.8625mmolATP/(gDCW\bullet h)$

And the GAM at dilution rate of 0.1 h^-1^ was computed as follows:

$$GAM=\frac{3.753}{80}\times18.5\times0.1\times1000mmol/(gDCW\bullet h)=86.7881mmolATP/(gDCW\bullet h)$$

In summary, the biomass equation of *Y. lipolytica* in this model is as follows:

(0.2815)13BDglcn[c] + (0.2471)ala-L[c] + (0.0482)amp[c] + (0.1464)arg-L[c] + (0.1647)asn-L[c] + (0.1647)asp-L[c] + (86.7881)atp[c] + (0.0368)cmp[c] + (0.0305)cys-L[c] + (0.0244)damp[c] + (0.024)dcmp[c] + (0.024)dgmp[c] + (0.0244)dtmp[c] + (0.0054)ergst[c] + (0.2013)gln-L[c] + (0.2013)glu-L[c] + (0.18)gly[c] + (0.2591)Chitin[c] + (0.0593)gmp[c] + (83.6890)h_2_o[c] + (0.0946)his-L[c] + (0.1342)ile-L[c] + (0.2684)leu-L[c] + (0.2135)lys-L[c] + (0.0704)mannan[c] + (0.0946)met-L[c] + (0.0013)pa_YL[c] + (0.0141)pc_YL[c] + (0.0103)pe_YL[c] + (0.1464)phe-L[c] + (0.1434)pro-L[c] + (0.0038)ps_YL[c] + (0.0125)ptd1ino_YL[c] + (0.1708)ser-L[c] + (0.02)so_4_[c] + (0.1861)thr-L[c] + (0.002)tre[c] + (0.3333)triglyc_YL[c] + (0.0305)trp-L[c] + (0.0946)tyr-L[c] + (0.0397)ump[c] + (0.061)val-L[c] + (0.0253)zymst[c] -> (86.7881)adp[c] + (86.7881)h[c] + (86.7881)pi[c]

**References**

1. Hong, Seung-Pyo, (Hockessin D, Sharpe, L P, et al. (2009) Optimized strains of Yarrowia Lipolytica for high eicosapentaenoic acid production. United States: E. I. DU PONT DE NEMOURS AND COMPANY, Wilmington, DE. pp. 69.

2. Schulze U (1995) Anaerobic Physiology of Saccharomyces Cerevisiae: Technical University of Denmark. 506 p.

3. Zvyagilskaya R, Andreishcheva E, Soares MIM, Khozin I, Berhe A, et al. (2001) Isolation and Characterization of a Novel Leaf-Inhabiting Osmo-, Salt-, and Alkali-Tolerant Yarrowia lipolytica Yeast Strain. Journal of basic microbiology 41: 289-303.

4. Groves DP, Oliver SG (1984) Formation of intergeneric hybrids of yeast by protoplast fusion of &lt;i&gt;Yarrowia&lt;/i&gt; and &lt;i&gt;Kluyveromyces&lt;/i&gt; species. Current Genetics 8: 49-55.

5. Ahsanul Islam M, Edwards EA, Mahadevan R (2010) Characterizing the Metabolism of Dehalococcoides with a Constraint-Based Model. PLoS Comput Biol 6: e1000887.

6. Chi Z, Yan K, Gao L, Li J, Wang X, et al. (2008) Diversity of marine yeasts with high protein content and evaluation of their nutritive compositions. Journal of the Marine Biological Association of the United Kingdom 88: 1347-1352.

7. Feist AM, Henry CS, Reed JL, Krummenacker M, Joyce AR, et al. (2007) A genome-scale metabolic reconstruction for Escherichia coli K-12 MG1655 that accounts for 1260 ORFs and thermodynamic information. Mol Syst Biol 3.

8. Barth G, Gaillardin C (1997) Physiology and genetics of the dimorphic fungus Yarrowia lipolytica. FEMS Microbiology Reviews 19: 219-237.

9. Papanikolaou S, Gortzi O, Margeli E, Chinou I, Galiotou-Panayotou M, et al. (2008) Effect of Citrus essential oil addition upon growth and cellular lipids of Yarrowia lipolytica yeast. European Journal of Lipid Science and Technology 110: 997-1006.

10. Papanikolaou S, Chatzifragkou A, Fakas S, Galiotou-Panayotou M, Komaitis M, et al. (2009) Biosynthesis of lipids and organic acids by Yarrowia lipolytica strains cultivated on glucose. European Journal of Lipid Science and Technology 111: 1221-1232.

11. Athenstaedt K, Jolivet P, Boulard C, Zivy M, Negroni L, et al. (2006) Lipid particle composition of the yeast Yarrowia lipolytica depends on the carbon source. Proteomics 6: 1450-1459.

12. Forster J, Famili I, Fu P, Palsson B脴, Nielsen J (2003) Genome-Scale Reconstruction of the Saccharomyces cerevisiae Metabolic Network. Genome Research 13: 244-253.

13. Thiele I, Palsson BO (2010) A protocol for generating a high-quality genome-scale metabolic reconstruction. Nat Protoc 5: 93-121.

14. Papanikolaou S, Aggelis G (2002) Lipid production by Yarrowia lipolytica growing on industrial glycerol in a single-stage continuous culture. Bioresource Technology 82: 43-49.
